# Developmental Instability in Incipient Colonies of Social Insects

**DOI:** 10.1371/journal.pone.0113949

**Published:** 2014-11-25

**Authors:** Thomas Chouvenc, Mathieu Basille, Hou-Feng Li, Nan-Yao Su

**Affiliations:** 1 Department of Entomology and Nematology, Fort Lauderdale Research and Education Center, University of Florida, Institute of Food and Agricultural Sciences, Fort Lauderdale, Florida, United States of America; 2 Department of Wildlife Ecology and Conservation, Fort Lauderdale Research and Education Center, University of Florida, Institute of Food and Agricultural Sciences, Fort Lauderdale, Florida, United States of America; 3 Entomology Department, National Chung Hsing University, Taichung City, Taiwan; Colorado State University, United States of America

## Abstract

Social insect colonies can provide homeostatic conditions that buffer the incidence of environmental fluctuations on individuals, which have contributed to their ecological success. *Coptotermes* (Isoptera: Rhinotermitidae) is a highly invasive termite genus and several species have important economic impact in many areas of the world. Mature *Coptotermes* colonies with millions of individuals can provide optimal environmental condition and nurturing capacity for the developing brood. However, it was previously suggested that contrary to mature colonies, incipient colonies may be exposed to critical stress, which may explain for the low success rate of establishment within the first year of the life of a termite colony. We here investigated the stress imposed on individuals of incipient colonies by comparing the developmental instability of individuals between incipient and mature colonies of two *Coptotermes* species, *C. formosanus* Shiraki and *C. gestroi* (Wasmann). We assessed the developmental instability by measuring the asymmetry of morphological traits from the head capsule of the soldier caste. Soldiers from incipient colonies of both species displayed strong asymmetrical traits in comparison to soldiers from mature colonies. We suggest that homeostatic conditions for optimal development are reached as the colony matures, and confirmed that the incipient colony remains a critical bottleneck where individuals are exposed to high developmental stress.

## Introduction

The developmental stability of an organism is reflected by its ability to produce an ‘ideal’ form under optimal conditions [1], [2]. During their development, organisms are exposed to stress of genetic and environmental origins which can result in individuals displaying morphologies that deviate from normality, mostly expressed in the breakdown of symmetry [2]. The measurements of asymmetrical traits have therefore been widely used as sensitive markers that can reveal stressful pressures during the early development of individuals [3]–[5].

Social insects can form large colonies with a protected nest structure that buffers external environmental changes, resulting in homeostatic conditions inside the nest [6]–[8]. The ability of social insect colonies to regulate the conditions of the inner nest has contributed to their success as they can easily adapt to a wide range of ecological niches [6], [9] and proved to be highly invasive in many cases [10]–[13] with seven social insect species among the 17 most invasive land arthropod species [14]. Studies on the developmental instability in social insects were mostly restricted to the effect of ploidy, inbreeding, heterozygosity and hybridization in ants and bees [15]–[19]. Only a few studies have looked at environmental cues as a source of developmental instability, including the effect of temperature on honey bees [20] and the effect of chronic exposure to metal pollutants in ant colonies [21]. In his study of ant exposure to pollutants, Rabitsch [21] mentioned that those in young colonies appeared to be more sensitive to metal exposure than ants from mature colonies.

In social insects, colonies are established during founding events, where the first brood is nurtured by the primary reproductive(s) and the first workers then take over nurturing tasks for the following broods [22], [23]. The rate of success of incipient colonies can be extremely low, owing to predation, competition, adverse environmental conditions, diseases, and the inability of the primary reproductive(s) to raise the first brood [22], [23]. Contrary to mature colonies, incipient colonies can therefore be at a critical bottleneck [24] where the small number of individuals, the limited nurturing capacity and the budding nest structure may not provide optimal conditions for individuals to develop properly, ultimately resulting in the failure for most young colonies to establish [22]–[25].

Termites (Isoptera) are social insects that present distinct caste polymorphism and polyethism [26], [27]. The genus *Coptotermes* (Rhinotermitidae) is the most economically important structural pest in tropical, subtropical and warm temperate areas of the world [12], [28], [29], as *Coptotermes* species can build large underground nest structures with millions of individuals [30], [31], causing serious damages to man-made structures. The two species *C. formosanus* Shiraki and *C. gestroi* (Wasmann) are the most successful termite invaders and have spread in several parts of the world [13], [29] with the help of human maritime transportations [32].

**Figure 1:**
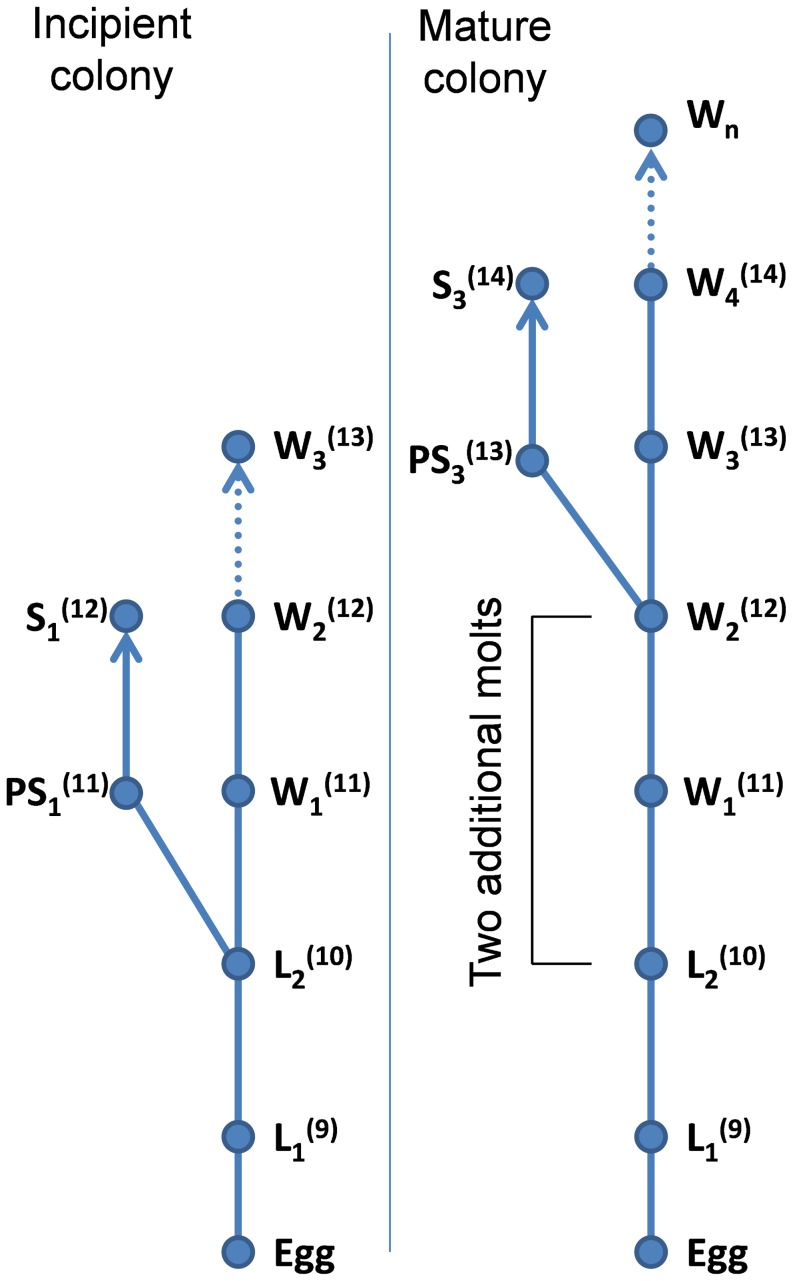
Developmental pathways for *C. formosanus* soldiers, modified from Chouvenc and Su [38]. L_1_, L_2_ = first and second instar larvae, W_1_, …, W_n_ = worker intars, PS_n_ = Presoldier from developmental pathways, S_n_ = Soldier from developmental pathways. Numbers in parentheses are the representative number of antennal segments of the given instar.

Termite colonies contain a specific soldier proportion [33] where a high developmental plasticity with complex epigenetic factors can be involved [34]–[36]. In addition, the developmental pathway for the production of soldiers is colony age-dependent in *Coptotermes* [37], [38]. Termite soldiers are technically a terminal caste in the sense that once molted into a soldier, it is a final molt, contrary to workers that keep molting until death [34], [38]. However, mature colonies produce soldiers that are derived from worker instars while in incipient colonies, soldiers are derived from the second instar larvae, resulting in a nanitic form [39] with two fewer molts (Fig. 1). The physiological constraints of the colony imposes an optimal soldier ratio at all time [40], [41], however young colony are resource limited and the investment in soldiers comes with a inherent cost [42]. Therefore, it was suggested that the accelerated development of nanitic soldiers was an efficient compromise for incipient colonies to quickly reach an optimal soldier ratio imposed by physiological constraints, while limiting the cost on the overall nurturing capacity of the colony [38], [42].

As nanitic soldiers are a characteristic of incipient colonies and are a direct product of a unique temporal condition, they are exposed to different environmental and physiological stress than mature colonies. We here propose to investigate the effect of the age of the colony on the developmental stability of soldiers under the hypothesis that individuals developing in incipient colonies are exposed to more intense stress than individuals in mature colonies. To this end, we evaluated the bilateral asymmetry of morphological traits of the head capsule of nanitic soldiers and soldiers from mature colonies in two termite species: *C. formosanus* and *C. gestroi*.

## Materials and Methods

### Ethic statement

No specific permits were required for the termite's collection from the field and for maintenance in laboratory. No endangered or protected species were involved in this study. All termites used for incipient colonies in this study were obtained from swarming events collected in Broward County (Florida, USA). Termites obtained from mature colonies were obtained from field colonies in the same area (10 km radius from GPS coordinates 26.08°N, 80.21°W) and maintained in laboratory for 1 to 3 months before use.

### Termite soldier terminology and morphology

Termites (Rhinotermitidae) are heterometabolous insects where workers molt throughout their lives [43]; however, the termite soldier caste is a terminal caste [34] therefore, although all soldiers are technically “fully developed,” for ease of description in this study, we used the term “nanitic soldier” for soldiers sampled from incipient colonies that displayed 12 antennal segments, and “mature soldier” for soldiers sampled from mature colonies that displayed 14 antennal segments (Fig. 1).

**Figure 2:**
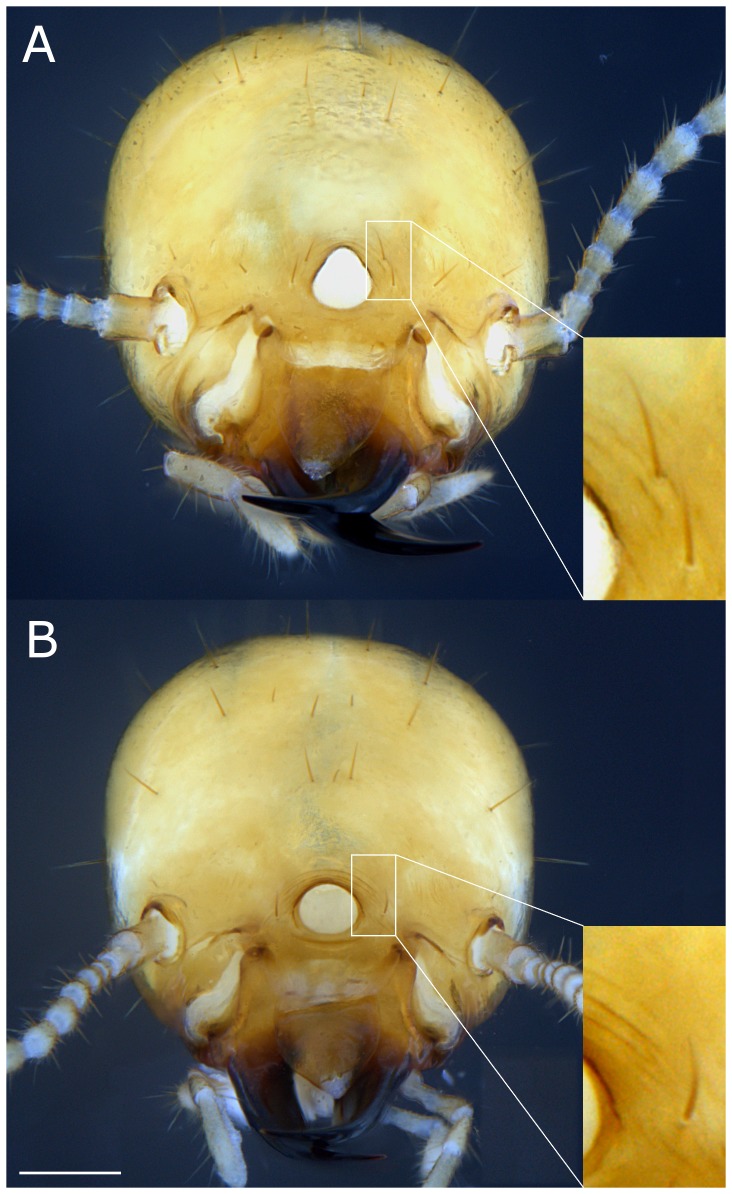
Frontal view of *Coptotermes* mature soldiers. A: *C. formosanus* displaying a double pair of setae around the fontanelle. B: *C. gestroi* displaying a single pair of setae at the base of the fontanelle. Bar = 200 µm.

Coptotermes soldiers have a characteristic fontanelle, an opening on the frontal part of the head from which *Coptotermes* soldiers can exude defensive secretions [44], [45]. In locations where both *C. formosanus* and *C. gestroi* are established [29], [46], [47], a simple morphological soldier characteristic is currently used to differentiate the two species [48]; a single pair of setae at the base of the fontanelle for *C. gestroi*, and two pairs of setae for *C. formosanus* (Fig. 2). This criterion has been reliably used when inspecting structures infested by a mature termite colony to determine which species was present [49]. In the current study, we investigated the variability of morphotypes in *C. formosanus* and *C. gestroi* by measuring the asymmetry of the pair(s) of setae around the fontanelle in nanitic soldiers and mature soldiers.

### *Coptotermes* incipient colonies foundation

Alates from dispersal flights of *C. formosanus* and *C. gestroi* were collected in Broward County (Florida, USA) during evenings between March and May 2013, using a light trap [50]. Collected alates naturally dropped their wings ( = dealates), and were stored overnight in vials containing a rolled piece of moist corrugated cardboard to be transported to the laboratory the next morning. Species identification of the dealates was done using the description given by [51]–[53]. Dealates were paired (one male and one female of the same species) and introduced into individual rearing units (37 ml vials, modified from [54], and fully described in [38]. Rearing units were stored at 28°C for 10 months and small amounts of water were occasionally added to vials that showed signs of dryness. After several months of development, successful incipient colonies (24 for *C. gestroi* and 18 for *C. formosanus*) produced 40–70 individuals with the presence of ≈10% soldiers.

### Soldier collection and measurements

Observations were performed by opening rearing units 185 d to 275 d after paring and sampling soldiers of both species, which were preserved in 85% ethyl alcohol before microscopic observation. All soldiers produced in this time frame were nanitic with 12 antennal segments and derived from second instar larvae, as described by [38]. A total of 100 nanitic soldiers were collected for each species. After nanitic soldiers were removed from incipient colonies, they were replaced within 45 d [42]. Observations of setae around the fontanelle were made on the microscope by setting individuals in hand sanitizer gel (63% alcohol), with the mandibles facing toward the microscopic lens to obtain a frontal view of the specimens. Because all observations were done with a frontal view of the soldiers, the “left” and “right” setae were determined from the observer point of view.

The standardized observation angle (frontal view) for all individuals allowed us to determine the number of setae for all 200 individual. In addition, the exact location of the point of origin of each seta was determined for 30 randomly selected nanitic soldiers presenting the most common morphotype observed in both species, i.e. a double pair of setae (see Results). Automontage (microscope Leica M205C coupled with a camera Leica DFC425) was used to obtain digital pictures, which were re-proportioned to a unique template using the fontanelle and the primary articulation point of both mandibles [55] for alignment. As a result, the coordinates of the point of origin of each seta could be mapped for all individuals in a standard referential plan (1 pixel≈1 µm). For comparison with the traits from mature soldiers, 100 soldiers per species from mature colonies were investigated following the same methodology and the location of setae was determined for 30 randomly selected mature soldiers that displayed the characteristic morphology of their species (3 colonies of origin for each species, 10 soldiers per colony).

### Counts and distributions of asymmetrical traits

In a first step, the overall number of setae by individual was compared in nanitic soldiers between the two species using a *t*-test (*n* = 100). The bilateral distribution of setae was then established for both species, with each possible combination of number of setae on each side of the fontanelle embedded in a left×right matrix. For each species, the symmetry was first tested using paired *t*-tests on the number of setae on the right *vs.* the number of setae on the left for each individual. The overall bilateral distributions of setae were then compared among species using a *χ* ^2^ test on all right-left combinations. Because many combinations had fewer than 5 individuals, we computed *p*-values by Monte-Carlo simulation. This protocol was repeated with mature soldiers from both species.

In a second step, we analyzed the locations of setae ([X_R_;Y_R_] for the right setae and [X_L_;Y_L_] for the left setae) from nanitic soldiers with the most common morphotype for each species, i.e. two pairs of setae (*n* = 30), and their mature counterparts (two pairs of setae for *C. formosanus*, only one pair for *C. gestroi*). We first computed core and global setae position areas using 50% and 95% contours of kernel densities for each type of setae (top-left, top-right, bottom-left and bottom-right). We used these contours to compare the areas and spatial overlaps of setae locations among soldiers morphotypes within each species (nanitic *vs.* mature) and between species (*C. formosanus* vs. *C. gestroi*). Two indexes were used to estimate distribution overlaps (see [56] for more details): PHR*_ij_*, which gives the probability to find setae from group *i* in the distribution of setae of group *j*, and the Bhattacharyya's affinity (BA), which gives a synthetic measure of similarity between the distributions of setae from the two groups.

### Analysis of fluctuating and directional asymmetries

We evaluated fluctuating and directional asymmetries using the framework of right-minus-left (R-L) frequency distributions [1], [57]. In order to compare right and left setae, we first computed the symmetrical location of the left setae on the right side using the vertical line passing through the center of the fontanelle as a reference: the X-coordinate (X_sL_) is then mirrored around the center of the fontanelle, while the Y-coordinate (Y_sL_) remains unchanged. We then computed individual R-L displacement vectors for each pair of setae as the vector with coordinates [X_R_ – X_sL_; Y_R_ – Y_sL_], i.e. the displacement on the X- and Y-axes from the symmetric left seta to the right seta. This bidimensional measurement (X and Y displacements) was finally reduced to a unidimensional measurement (hereafter simply “R-L displacement”) as the projected coordinate on the axis of greatest R-L displacement (i.e. the axis passing through the average displacement vector, see Material S1). With this new basis, any deviation from the ideal fluctuating asymmetry (characterized by a normal distribution with a mean of 0, i.e. no displacement in average) may indicate genetically or environmentally-induced developmental noise [57]. We assessed deviation from normality using Kolmogorov-Smirnov tests and differences from a null mean, i.e. directional asymmetry, using *t*-tests. Finally, comparisons of means (using *t*-tests) and variances (using *F* tests) of R-L distributions evaluated the relative importance of fluctuating and directional asymmetries among soldiers morphotypes (nanitic vs. mature) and between species (*C. formosanus vs. C. gestroi*). All statistical analyses were performed using R 3.0 [58] and the package adehabitatHR for estimating kernel contours [59]. The full description of the asymmetry analysis is available in the Material S1.

## Results

### Setae counts in *C. formosanus* and *C. gestroi*

**Figure 3:**
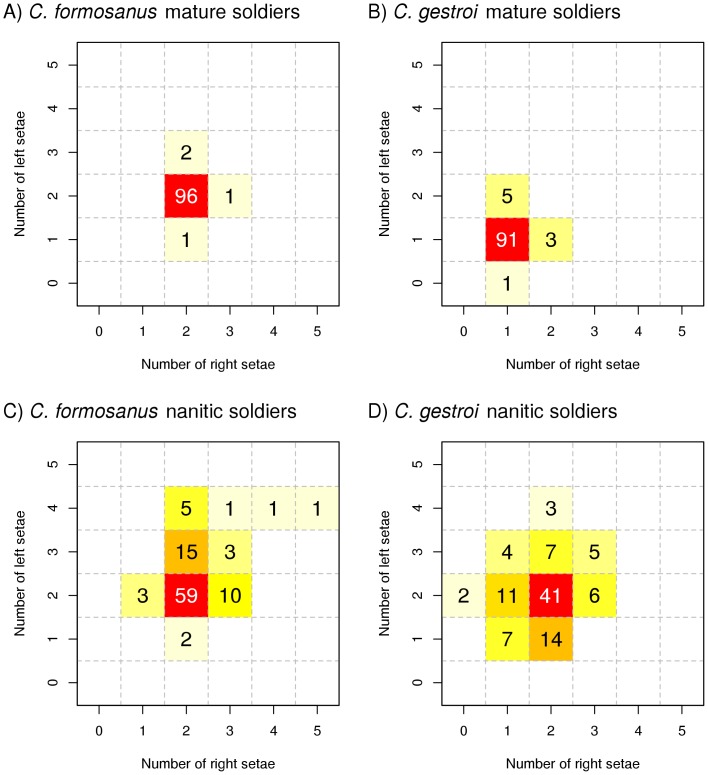
Number of nanitic soldiers displaying a given morphotype (number of setae on each side of the fontanelle). n = 100 individuals per species and per age group. Left and right positions are from the observer point of view from a frontal display of individuals.

The number of setae around the fontanelle of nanitic soldiers was variable in both species, with a wide range of combinations, going from 0 to 5 setae on each side of the fontanelle, with a total of 2 to 9 setae in extreme cases (Fig. 3, see Material S2 for morphotypes examples). The overall number of setae in nanitic soldiers was significantly higher in *C. formosanus* than in *C. gestroi* (4.48 vs. 3.86, *t* = 4.441, df = 196.8, *p*<0.001). For both species, the most representative morphotype of nanitic soldiers was a double pair of setae around the fontanelle (59% and 41% for *C. formosanus* and *C. gestroi*, respectively); as such, *C. gestroi* nanitic soldiers did not predominantly display the characteristic morphotype of their own species as seen in mature soldiers. However, the bilateral distributions were significantly different between the two species (χ^2^ = 37.72, *p*<0.001, Fig. 3). While the distribution was slightly skewed to the left in both species (+0.16 seta on the left in average for both species), the difference in the number of setae between right and left of the fontanelle was significant for *C. formosanus* only (paired *t*-test, *C. formosanus*: *t* = 2.264, df = 99, *p* = 0.026; *C. gestroi*: *t* = 1.883, df = 99, *p* = 0.063).

Mature soldiers of *C. formosanus* displayed the characteristic double pair of setae for most observed individuals (>95%). However, few individuals developed one extra or one fewer seta around the fontanelle (Fig. 3). Similarly, mature soldiers of *C. gestroi* displayed the characteristic single pair of setae for most individuals (>90%) but some individuals developed one or two extra setae around the fontanelle. Because this study aims to compare the asymmetry between mature and nanitic soldiers, only the mature soldiers displaying the characteristic morphotype of their species were used for the symmetry analysis (*n* = 30).

### Setae distributions in nanitic and mature soldiers

**Figure 4:**
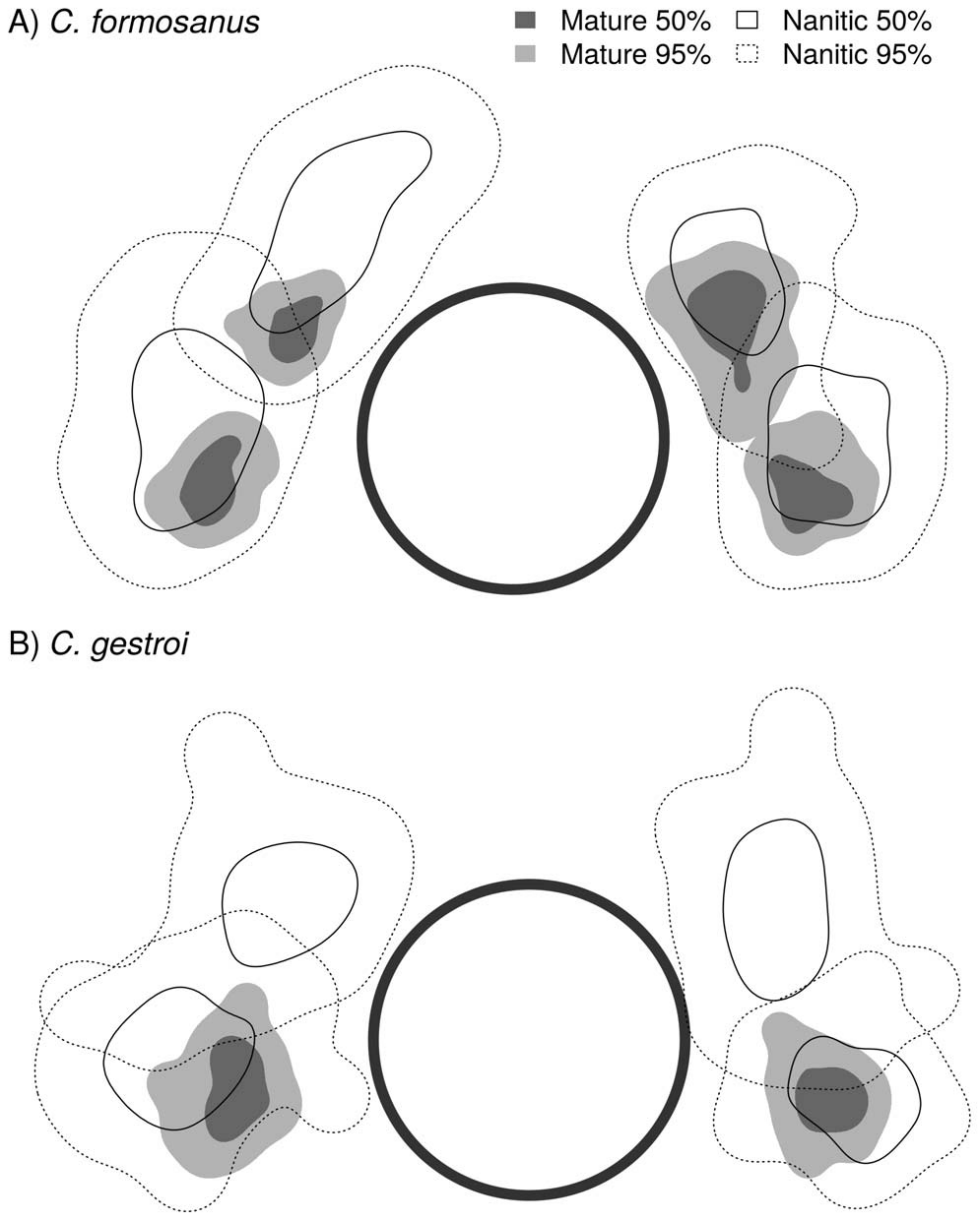
Global distributional areas using 50% (dark grey and solid line) and 95% (light grey and dotted line) contour of kernel densities for each type of setae (top-left, top-right, bottom-left and bottom-right) in mature and nanitic soldiers of A) *C. formosanus* and B) *C. gestroi* (30 individuals per category). The central circle represents a standardized fontanelle (diameter≈100 µm).

In *C. formosanus*, the distribution of setae around the fontanelle (top-left, top-right, bottom-left, bottom-right) was much more scattered for nanitic than for mature soldiers (mean 50% and 95% distribution areas were 4.8 times larger for nanitic soldiers, Fig. 4A).The overlap metrics indicated a mild similarity in setae distribution areas between nanitic and mature soldiers, which shared less than two thirds of their spatial distributions (BA = 0.619). However, more than 99% of the mature setae locations were included in the global (95%) distribution area of nanitic setae, while only 34% of the nanitic setae locations were included in the global distribution area of mature setae. The distribution areas were noticeably wider on the right than on the left for both top and bottom setae in mature soldiers (50% and 95% areas were 1.6 times larger on the right). However, the opposite was observed for nanitic soldiers: 50% and 95% areas were 1.3 times larger on the left.

In *C. gestroi*, the distribution of setae around the fontanelle was also much more scattered for nanitic than for mature soldiers (mean 50% and 95% distribution areas of bottom setae were 3 times larger for nanitic, no possible comparison for top setae). The overlap between mature and nanitic soldier setae distributions also strongly indicated that the unique pair of setae in mature soldiers corresponds to bottom setae of nanitic individuals (Fig. 4B).The similarity in distribution area between nanitic and mature soldier setae distribution areas was of the same order than for *C. formosanus* (BA = 0.700), and while 97% of the mature soldier setae locations were included in the global (95%) distribution area of nanitic soldier setae, only 45% of the nanitic soldier setae locations were included in the global distribution area of mature soldier setae. The distribution areas were consistently wider on the left than on the right for bottom setae in both nanitic and mature soldiers (50% and 95% areas were 1.3 times larger on the left). However, distribution areas were slightly larger for the top-right setae than for the top-left setae in nanitic soldiers (50% and 95% areas were 1.1 larger on the right).

### Setae asymmetry in nanitic and mature soldiers

**Figure 5:**
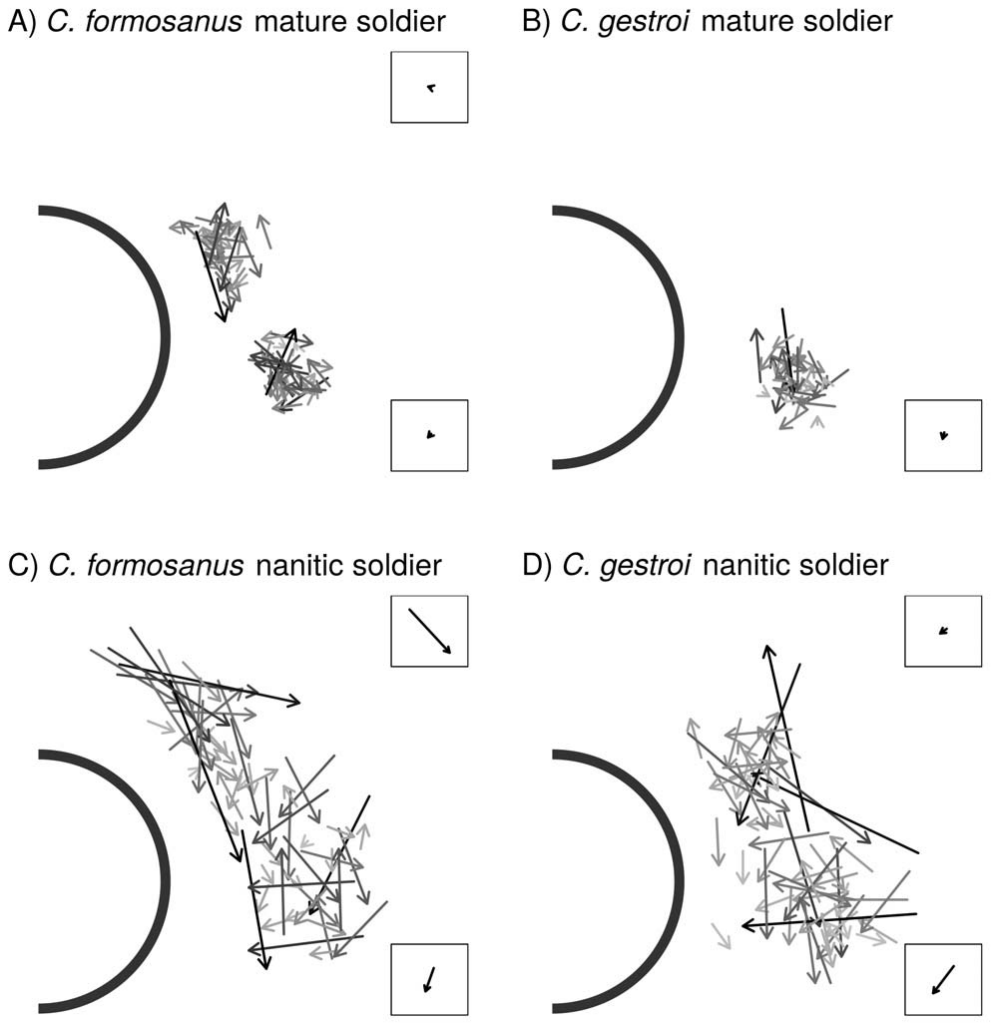
Fluctuating and directional asymmetries represented using individual Right-Left displacement vectors for each pair of setae and each morphotype (n = 30 per morphotype). Fontanelle diameter≈100 µm. Each arrow represents the asymmetry for a pair of setae, with the color intensity indicating the relative Right-Left displacement (the darker the arrow, the bigger the relative displacement). The top and bottom average displacements are displayed in the top and bottom corners respectively for each morphotype.

In *C. formosanus* mature soldiers, both distributions of top and bottom setae R-L displacements followed a normal distribution (top: *D* = 0.108, *p* = 0.839; bottom: *D* = 0.119, *p* = 0.745). Both means were not significantly different from zero, with an average R-L displacement of 1.3 µm for top setae (*t* = 0.607, df = 29, *p* = 0.274) and 2.2 µm for bottom setae (*t* = 1.063, df = 29, *p* = 0.148), indicating no directional asymmetry in the setae distribution of mature *C. formosanus* soldiers (Fig. 5A). Both distributions of top and bottom setae R-L displacements for *C. formosanus* nanitic soldiers also followed a normal distribution (top: *D* = 0.087, *p* = 0.961; bottom: *D* = 0.122, *p* = 0.718), but the means were significantly different from zero, with an average R-L displacement of 28.0 µm for top setae (*t* = 6.285, df = 29, *p*<0.001) and 12.0 µm for bottom setae (*t* = 2.783, df = 29, *p* = 0.005), indicating a directional asymmetry towards the bottom-right (top setae) and towards the bottom left (bottom setae; Fig. 5C). The variances of the distributions of both top and bottom setae R-L displacements were more than 4 times larger in nanitic than in mature *C. formosanus* soldiers (top: *F* = 4.210, df_1_ = 29, df_2_ = 29, *p*<0.001; bottom: *F* = 4.358, df_1_ = 29, df_2_ = 29, *p*<0.001), indicating a larger fluctuating asymmetry in nanitic soldiers than in mature soldiers.

In *C. gestroi* mature soldiers, the distribution of setae R-L displacements followed a normal distribution (bottom: *D* = 0.094, *p* = 0.933), with a mean not significantly different from zero (average R-L displacement = 3.3 µm, *t* = 1.299, df = 29, *p* = 0.102; Fig. 5B). Both distributions of top and bottom setae R-L displacements for *C. gestroi* nanitic soldiers also followed a normal distribution (top: *D* = 0.080, *p* = 0.983; bottom: *D* = 0.108, *p* = 0.840), but only bottom setae were in average significantly different from zero, with an average R-L displacement of 3.8 µm for top setae (*t* = 0.942, df = 29, *p* = 0.177) and 16.3 µm for bottom setae (*t* = 4.883, df = 29, *p*<0.001), indicating a directional asymmetry towards the bottom left. The variances of the distributions of bottom setae R-L displacements between nanitic and mature *C. gestroi* soldiers suggested a stronger fluctuating asymmetry in nanitic soldier but was not significant (bottom pair only, *F* = 1.765, df_1_ = 29, df_2_ = 29, *p* = 0.066) (Fig. 5D).

The comparison of variances of the R-L displacements between *C. formosanus* and *C. gestroi* nanitic soldiers (top: *F* = 1.25, df_1_ = 29, df_2_ = 29, *p* = 0.552; bottom: *F* = 1.686, df_1_ = 29, df_2_ = 29, *p* = 0.166) and mature soldiers (bottom pair only, *F* = 0.683, df_1_ = 29, df_2_ = 29, *p* = 0.309) did not reveal any difference in fluctuating asymmetry. However, the comparison of the average R-L displacements highlighted a greater directional asymmetry for top setae in *C. formosanus* nanitic soldiers as compared to *C. gestroi* nanitic soldiers (difference of 24.3 µm, *t* = 4.057, df = 57.29, *p*<0.001), but not for bottom setae (difference of 4.2 µm, *t* = −0.775, df = 54.45, *p* = 0.442).

## Discussion

Our results showed that the number, the distribution, and the symmetry of the setae around the fontanelle of nanitic soldiers differed from mature soldiers in both *C. gestroi* and *C. formosanus*. First, the number of setae around the fontanelle of nanitic soldiers was highly variable with the presence of extreme morphotypes, while most mature soldiers (91–96%) displayed a number of setae representative of their own species (single pair for *C. gestroi*, double pair for *C. formosanus*). Second, the point of origin of each seta was spread over a wider area for nanitic soldiers in comparison to mature soldiers. Finally, directional and fluctuating asymmetry were much more pronounced in nanitic soldiers, as mature soldiers displayed relatively symmetric traits. These three observations all support our hypothesis that soldiers produced from incipient colonies have a stronger developmental instability than soldiers produced from mature colonies, which confirms that developing individuals in incipient colonies are exposed to a stress that is stronger than in mature colonies.

Incipient colonies have a reduced nurturing capacity with little access to resources, a minimal nest structure that may not buffer external environmental changes as efficiently as nests from mature colonies would, and soldiers are produced from an accelerated developmental pathway that can impose a unique physiological stress on these individuals. Nanitic soldiers can therefore be considered to have a “premature” morphology for which the development was accelerated in a resource-constrained environment, resulting in abnormal morphotypes, i.e. variable number and position of setae around the fontanelle. However, because the accelerated development, the reduced homeostatic conditions and the reduced nurturing capacity cannot be separated in incipient colonies (confounding factors), it is challenging to determine the extent of the contribution of each factors on the developmental instability we observed in nanitic soldiers. We also note that incipient colonies where raised in the laboratory, which is a more stable environment than the field. We suggest that incipient colonies in the field may be exposed to greater stress and therefore, the strong asymmetry we observed in nanitic soldiers may actually be even more pronounced in field incipient colonies. If the survival of the incipient colony depends on its ability to cope with such stress, we would expect field colonies to express higher development instability and lower survival than laboratory incipient colonies.

The fluctuating asymmetry of setae that we observed around the fontanelle of mature soldiers was weaker than for nanitic soldiers in both species. This result supports that the stress imposed on mature colonies is not as strong as in incipient colonies or that mature colonies are better equipped to buffer such stress, as the abundance of resources, the homeostatic environment, and the additional molts allow for less stress during the development of individuals. All termites used in this study were collected in South Florida, a location where both species were recently introduced (1980s–1990s), possibly with single introductions, presumably resulting in a high degree of inbreeding [60]–[62]. As the asymmetry in individuals can be the product of the interaction between the environmental stress and the genetic makeup of each individuals, especially in situation of inbreeding [1], [63], we here suggest that the strong asymmetry in nanitic soldiers of *Coptotermes* can be the consequence of both the developmental stress inherent to incipient colonies and high degree of inbreeding, while the weak asymmetry in mature soldiers may mostly be the result of inbreeding alone, or simply reflect the natural variability of this trait.

The role of the setae around the fontanelle in *Coptotermes* soldiers remains unclear. Overall, there was a lack of conservation in the position and number of the setae among nanitic soldiers of the two species. However, the fact that the “two-pair” of setae morphotype was displayed predominantly in nanitic soldiers of both species, but was maintained in *C. formosanus* mature soldiers only, may imply that it could be a pedomorphic trait, or a vestigial trait inherited from a common ancestor. Vestigial traits tend to display a higher fluctuating asymmetry than functional traits [64], thus the particularly stressful developmental conditions in incipient colonies may greatly amplify the instability of such trait in nanitic soldiers. To conclude, social insects provide unique opportunities to test hypotheses using morphological traits owing to their coloniality and their genetic homogeneity [65]; however, this study showed that the age of the colony can be an important factor on the developmental stability of individuals and future morphological studies using social insects may need to take into consideration such a factor.

## Supporting Information

Material S1 **Full description of the asymmetry analysis.**
(PDF)

Material S2 **Examples of extreme setae distribution in** ***C. formosanus*** **and** ***C. gestroi*** **nanitic soldiers.**
(PDF)
