# Supplementary material for: Developmental Instability in Incipient Colonies of Social Insects
Source: PLoS One. 2014 Nov 25;9(11):e113949. doi: 10.1371/journal.pone.0113949 (PMC4244189; doi:10.1371/journal.pone.0113949)
Supplement: Material S1 — Full description of the asymmetry analysis. (PDF) [file pone.0113949.s001.pdf]

# Supplementary material S1 "Developmental instability in incipient colonies of social insects"

**Asymmetry analysis**

Thomas Chouvenc, Mathieu Basille, Hou-Feng Li, Nan-Yao Su

October 29, 2014

## Introduction

In this document, we detail the asymetry analysis performed on nanitic and mature soldiers of *C. formosanus* and *C. gestroi*. Our aim was to compare the positions of left and right setae, using a R-L (right-minus-left) perspective, following a classical asymmetry approach, such as advocated by [57], to disentangle between fluctuating and directional asymmetry.

The analysis only focused on the $2 \times 2$ nanitics, i.e. the most common phenotype for the two species. They were compared to adults of both species. Note that all adults in *C. formosanus* have 2 pairs of setae, while all adults in *C. gestroi* have only one. In this document, we focused with details on the nanitic soldiers from *C. formosanus*, with the specific case of top setae, being understood that the analysis was identical for bottom setae, as well as for mature soldiers and *C. gestroi* termites.

## Displacement vectors

To be able to compare the location of right and left setae, a straightforward approach is to "mirror" the left setae on the right side, and then directly compare their location. The symmetric coordinate was calculated in relation to the fontanelle (and precisely, its center), as the coordinate of the center (here, 229 pixels) plus the difference between the center and the original coordinate of the seta:

$$x_{sym} = x_{center} + (x_{center} - x_{ori})$$

$$x_{sym} = 2 \times x_{center} - x_{ori}$$

From this, we computed the displacement vectors, i.e. the vectors that connect the left setae to the (symmetric) right setae (R-L vectors). The coordinates of the displacement vectors were thus defined as a measure of displacements on the X-axis and on the Y-axis, as can be seen on the figure below (the circle represents the fontanelle, and each insert in the top-right and bottom-right corners indicates the average top, and bottom, average displacement vector, respectively):

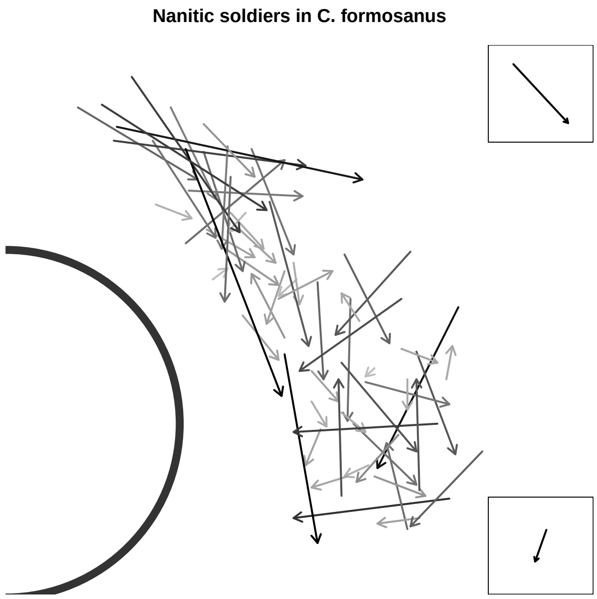


# Reducing the displacement vectors to one dimension

Classical asymmetry analyses relies on the comparison of a single measurement, e.g. the length of wings or limbs. In our case, each location was characterized by its X and Y coordinates, i.e. a bivariate measurement. In order to reduce this vector of coordinates to a single measurement, we projected each displacement vector on the line of greatest displacement, i.e. the line defined by the average displacement vector. The resulting one-dimensional vectors were then suitable for analysis.

We illustrate here this approach with the top setae. The following plot shows all displacement vectors centered on their origin (left seta), with the average displacement vector in red (the second dotted red vector is an orthogonal vector of the same lenght, which defines, together with the first one, an Euclidean space).

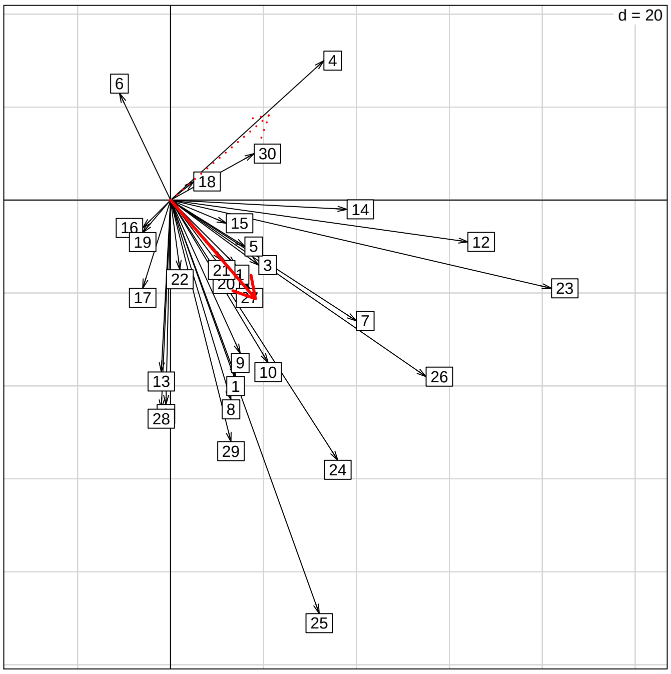


We then used these two vectors as a basis for the rotation of the whole data set. We rotated the cloud of points so that the line of greatest displacement was aligned on the X-axis, and the second orthogonal vector was aligned on the Y-axis. To do this, we actually projected all displacement vectors on these two vectors using a matrix product. We also projected these two vectors on themselves, which resulted in vectors with one coordinate equal to zero and the other one equal to the vector's norm. The result can be seen in the following, rotated, plot:

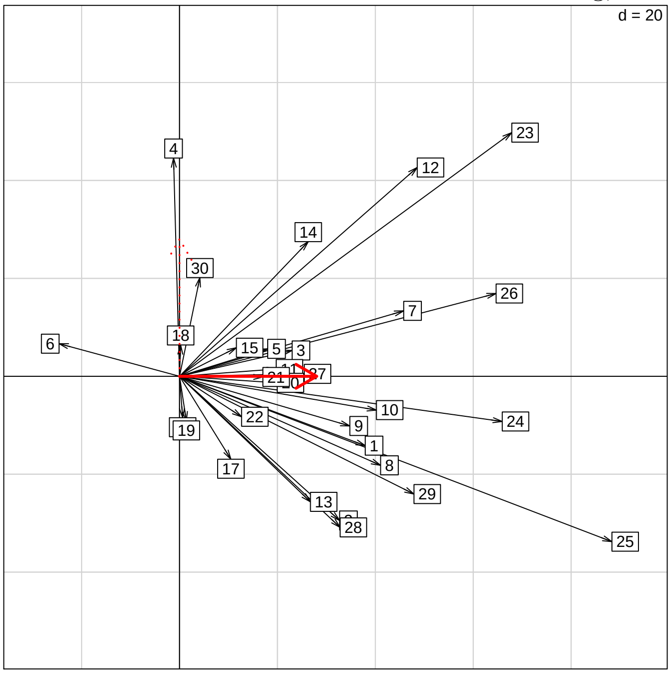


The coordinates of each vector on the X-axis were then used as a single measurement of

asymmetry.

## Testing the asymmetry

We first compared the coordinates on the line of greatest displacement to a normal distribution with the same mean and standard deviation, to assess the normality of the observed distribution. In a second step, we compared it to a normal distribution with mean zero and the same standard deviation (theoretical distribution), to assess the asymmetry:

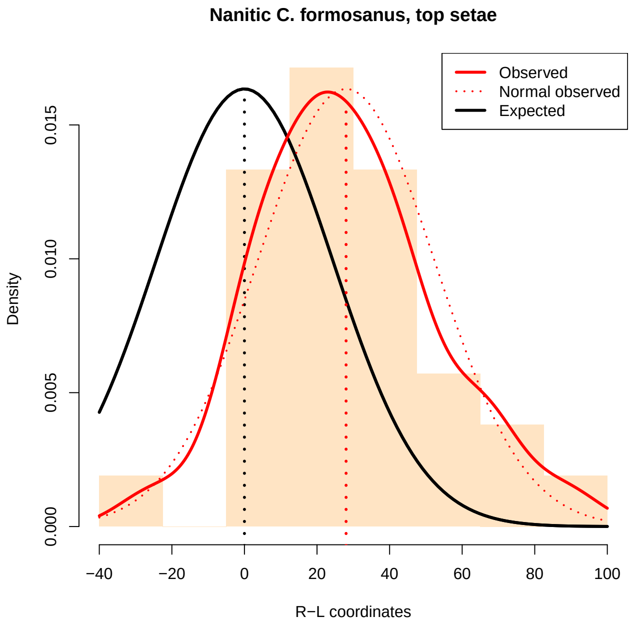


A non-parametric Kolmogorov-Smirnov test allowed us to test the difference between the observed distribution and a theoretical normal distribution, and actually highlighted a non-departure from normality ($D = 0.087$, $p = 0.961$). We thus directly tested the mean with a one-sided $t$-test, which highlighted a significant difference with 0 ($t = 6.285$, df $= 29$, $p < 0.001$). In other words, the displacement of the top setae towards the bottom-left as seen on the figure above was statistically significant.

**The whole procedure (displacement vectors, rotation and asymmetry tests) was then repeated for all morphotypes.**

## References

[57] Palmer, A. R. and Strobeck, C. (1992). Fluctuating asymmetry as a measure of developmental stability: implications of non-normal distributions and power of statistical tests. *Acta Zoologica Fennica*, 191:57–72.
