# Supplementary figures and images for: Developmental Instability in Incipient Colonies of Social Insects
Source: PLoS One. 2014 Nov 25;9(11):e113949. doi: 10.1371/journal.pone.0113949 (PMC4244189; doi:10.1371/journal.pone.0113949)

*C. formosanus*  
Nanitic soldier  
5-4

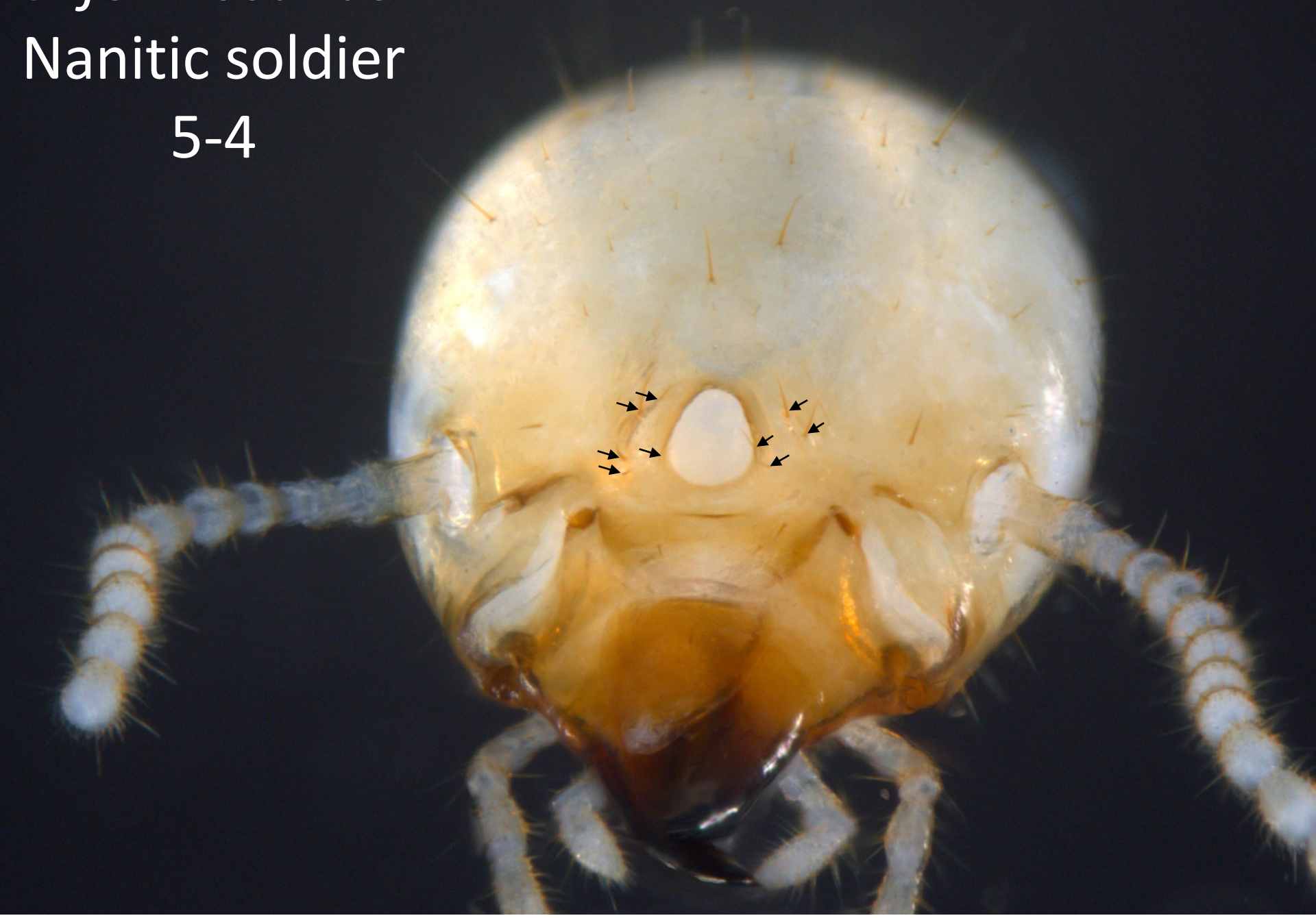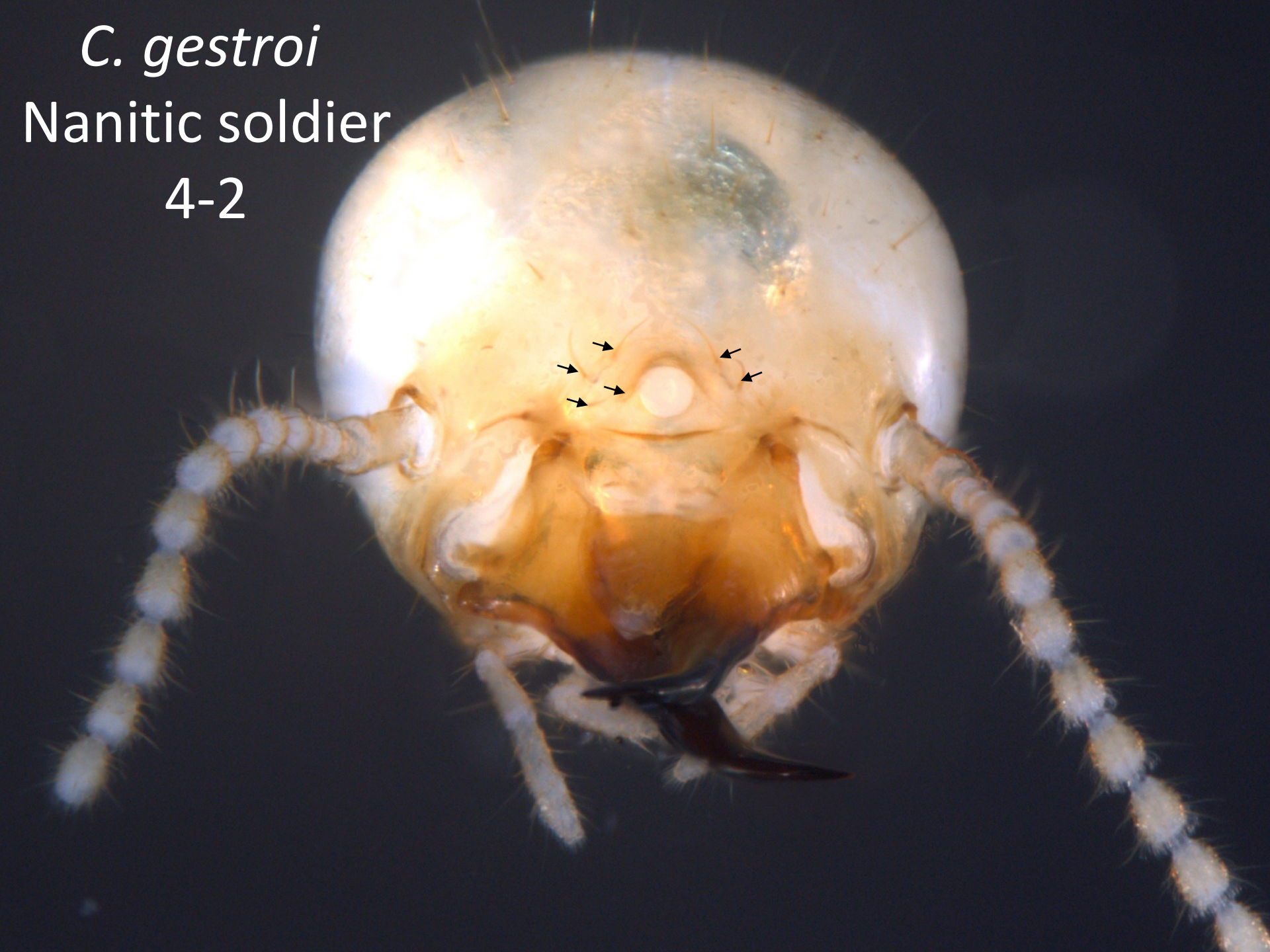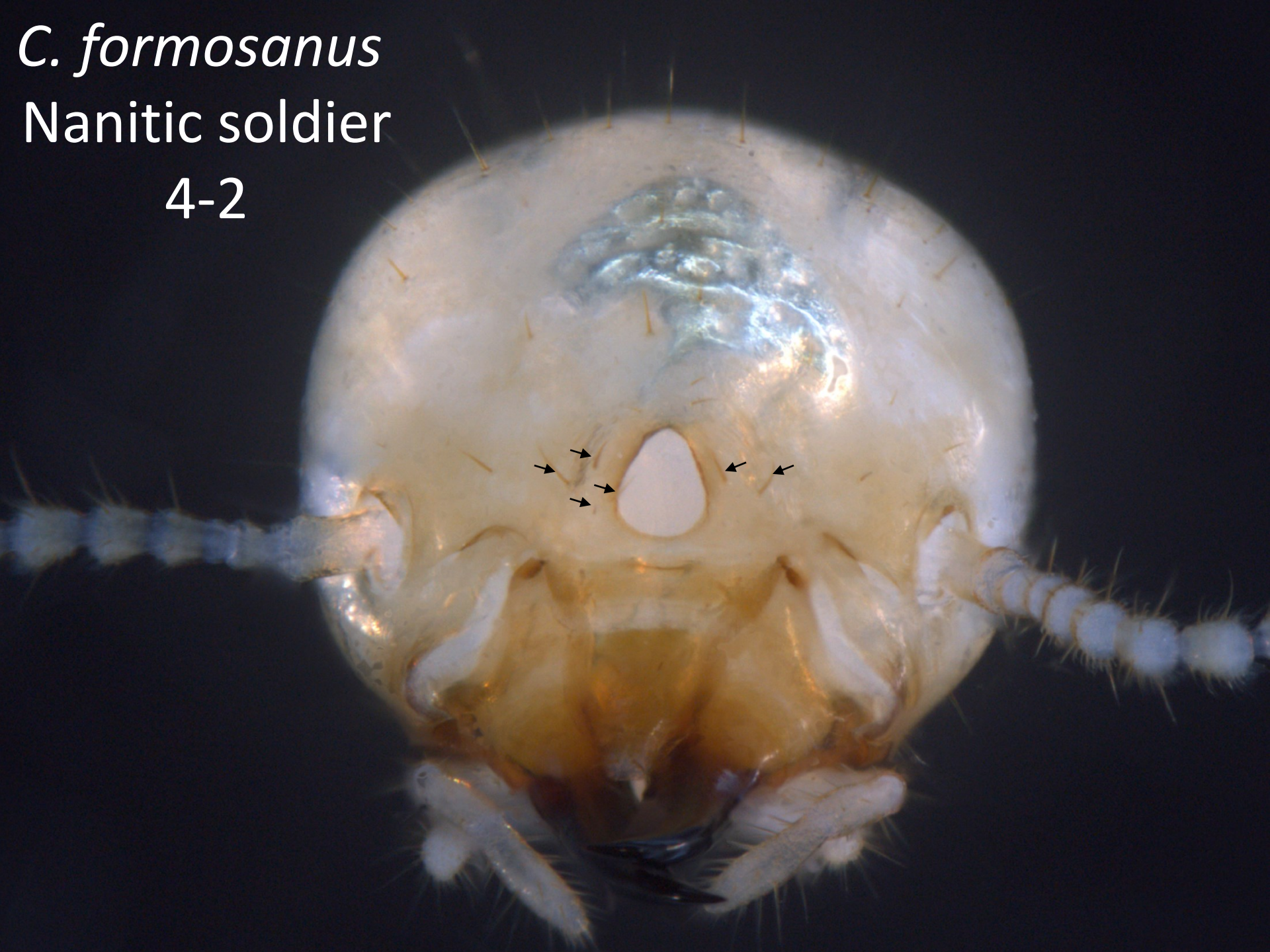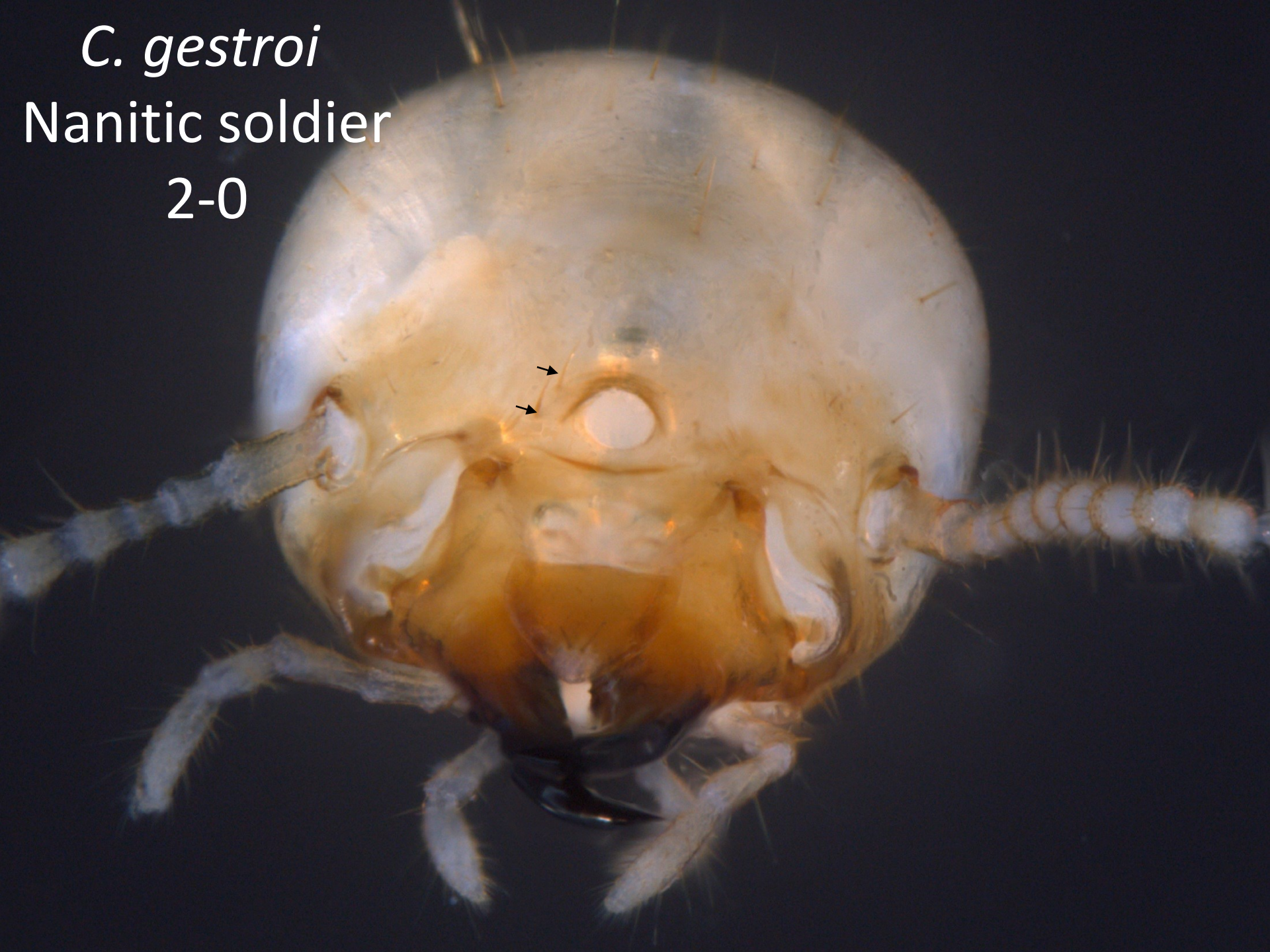

Supplement: Material S2 — Examples of extreme setae distribution in C. formosanus and C. gestroi nanitic soldiers. (PDF) [file pone.0113949.s002.pdf]
